# Informal Caregivers’ Experiences of an Online Support Program: Qualitative Study Using an Abductive Approach Focusing on Scaling Up Use

**DOI:** 10.2196/77576

**Published:** 2025-11-27

**Authors:** Hanna Allemann, Ingela Thylén, Frida Andréasson, Anna Strömberg

**Affiliations:** 1 Department of Health, Medicine and Caring Sciences Linköping University Linköping, Östergötland Sweden; 2 Department of Cardiology Linköping University Linköping, Östergötland Sweden; 3 Department of Social Work Linnaeus University Kalmar Sweden

**Keywords:** abductive approach, digital health engagement model, heart failure, implementation, informal caregivers, online support, digital health, health engagement, DIEGO, mobile phone

## Abstract

**Background:**

Informal caregivers of persons with chronic conditions such as heart failure have a crucial role in providing support. They often provide this help and care without formal training or financial compensation. Their situation can be experienced as demanding and complex, and caregivers have expressed a need for support. In response to their needs, an online support program was co-designed with and for caregivers. The co-design process ensured that the content is relevant to their needs and preferences. However, relevance alone does not guarantee that the program will be feasible or perceived as helpful in caregivers’ everyday lives.

**Objective:**

This study aimed to explore the experiences of informal caregivers of persons with heart failure who had access to an online support program and to identify facilitators and barriers in order to reflect on implementation strategies for scaling up use.

**Methods:**

Participants in this study were included from a multicenter randomized controlled trial in which the co-designed support program is being evaluated. This study used an abductive approach to explore caregivers’ experiences and to identify barriers, facilitators, and implementation strategies. Fifteen informal caregivers participating in the randomized controlled trial were interviewed for this purpose. The interviews were analyzed using qualitative content analysis, and the findings were subsequently mapped onto the Digital Health Engagement Model (DIEGO).

**Results:**

This study identified caregivers’ experiences of engaging with the support program, as well as factors influencing their use of the online content. Drawing on these experiences and applying DIEGO, reflections on tailored implementation strategies were formulated. These strategies emphasize the importance of active outreach to raise awareness of the support program, securing endorsements from relevant sources, and the continuous adaptation of the intervention to enhance its feasibility and relevance in caregivers’ everyday lives.

**Conclusions:**

A modified version of DIEGO proved valuable for identifying implementation strategies based on caregivers’ experiences, with the aim of scaling up use of the support program. Furthermore, DIEGO supported the identification of factors that may provide valuable insight into the implementation process, both in clinical practice and in research.

## Introduction

Informal caregivers provide nonprofessional (often unpaid) support, help, and care to persons with chronic conditions [1] and constitute the majority of long-term care providers in Europe [2,3]. It is anticipated that informal caregivers will assume an increasingly significant role in providing care, which can at times be complex, and they may find themselves unprepared for this responsibility [4]. Heart failure (HF) is a common chronic condition with poor prognosis [5,6], in which caregivers provide support in relation to an unpredictable illness trajectory [7,8]. The illness trajectory encompasses changes occurring both gradually and occasionally abruptly [6]. Caregivers’ experiences range from viewing informal care as a natural part of life [9] to finding it burdensome [10] or potentially rewarding [11]. Caregivers of persons with HF have expressed a need for knowledge and support [7,8,12,13], and research shows that they are increasingly turning to the internet for this [14], a trend that intensified during the COVID-19 pandemic [15]. Evidence has confirmed the use of online interventions to support caregivers [16-18]. Even so, there may be a fear that online solutions might replace valuable direct interactions with health care professionals [19]. Moreover, caregivers may not always recognize that they are in a caring role, and therefore may not seek support for themselves [20].

Our research group has co-designed an online support program with and for caregivers of persons with HF. The co-design process and the program have been previously described [21]. By involving caregivers in the co-design, we ensured that the content was relevant in relation to various caregiving trajectories, which have been described as an important facilitator for engagement in online interventions [22]. Further, the design and content were carefully considered to accommodate different literacy levels and promote inclusive language. Additionally, professionals and researchers provided trustworthy and evidence-based content, which is an important aspect of providing useful online solutions [19]. One factor we also considered was the potential for added burden on health care when scaling up access and use, which may be important as health care personnel can fear that online solutions add to the workload [23]. This affected the choice of platform and meant that we did not include the opportunity for caregivers to interact with health care through access to the support program, even though this was expressed as a desired feature. The co-design process also involved further trade-offs in relation to caregivers’ wishes and needs, and we did not provide caregivers with the opportunity to interact with other caregivers through the program. We know that this is a common and potentially important feature in supporting caregivers [24,25]. The trade-offs made were related to the technical constraints of the chosen platform and the wish to minimize the use of health care resources to support a broad implementation and sustainability. The program is hosted on 1177 (Inera), the well-integrated national health portal in Sweden [26], which offers a “secure system” considering personal data. This is a strength, since insecure systems have been raised as an issue that could pose a barrier to engaging with online solutions [19,23]. Moreover, 1177 aligns with current health care processes, which could be crucial for scaling up access and for sustainability [19,27]. It also fits into the contemporary focus on digitalizing health care to meet challenges related to scarce resources [28,29]. Additionally, the program may be considered “timely” because there are both international and national governmental initiatives with the intent to put caregivers, more obviously, on the agenda within all domains of health care and social care. This becomes clear not least through the launch of “caregiver strategies” on both national and European levels [30,31].

This study focuses on factors for scaling up the use of the support program across all health care regions in Sweden, as it is not self-evident that all regions have access to all content in the platform, despite it being a national health portal. The scaling up will have the potential to strengthen equal care, which is important to strive for both in Sweden and internationally. Therefore, this study aimed to explore the experiences of informal caregivers of persons with HF who had access to an online support program and to identify facilitators and barriers in order to reflect on implementation strategies for scaling up use. Tailoring strategies based on empirical data may improve chances of successful implementation [32].

## Methods

### Study Design

This study used an abductive approach inspired by Eriksson and Lindström [33], Graneheim et al [34], and Graneheim and Lundman [35] to describe caregivers’ experiences and identify barriers and facilitators to reflect on possible implementation strategies using The Digital Health Engagement Model (DIEGO) [36]. DIEGO is underpinned by 2 empirical theories: the normalization process theory (NPT) [37,38] and the burden of treatment theory (BOTT) [39]. NPT focuses on describing how actions become routine and part of people’s everyday lives, and it was used as the foundation for developing DIEGO. BOTT was developed to explain, for example, adherence and sustainability to treatment by considering a person’s “capacity for action,” and “the work that healthcare systems pass on to patients and their relational networks.” BOTT also draws on NPT and its constructs when explaining the “work” that patients and their network undertake in relation to illness. In DIEGO, BOTT was used “as a lens” to inform recommendations that support engagement with digital interventions. According to DIEGO, 4 main processes will affect a person’s decision and actions to adopt a digital health intervention, in our case, to engage in an online support program. These processes are “making sense of a digital health intervention,” “considering the quality of a digital health intervention,” “gaining support for enrolling in a digital health intervention,” and “registering for a digital health intervention.” Each process comprises subcomponents with identified barriers and facilitators, which further elucidate factors that may ultimately influence the use of digital interventions. We modified the specified barriers and facilitators within the subcomponents of the model to suit our specific intervention.

### Data Collection and Settings

The support program described in the background is being evaluated in an ongoing multicenter randomized controlled trial to test its efficacy on “preparedness for caregiving” (ClinicalTrials.gov NCT04885465, registered May 9, 2021). Participants in the intervention group have 3-month access to the online support program, during which they receive emails with instructions, as well as telephone calls and text messages to promote use. The control group is offered the intervention at the end of the study (waiting list).

Informal caregivers from the intervention group were consecutively invited for an individual interview. The first 9 informal caregivers were excluded because they had already been interviewed with a focus on feasibility. For this study, the subsequent 19 informal caregivers were asked to participate and 15 agreed. Participants were contacted via telephone and asked if they wanted to participate in an interview between 20-115 (median 30) days after gaining access to the online support program, and asked if they wished to participate in an interview. They were informed about this study and, if interested, received written information and were given time to consider their participation. If they agreed to participate, a time and a place for the interview were arranged. Participants could choose to be interviewed face-to-face (n=9), via video meeting (n=5), or by phone (n=1).

Semistructured interviews were conducted by the first author, HA, between August and October 2022. The interview guide (Multimedia Appendix 1) had been pilot tested to ensure its comprehensibility. No adjustments were made, and the pilot was therefore included. Interviews lasted between 12 to 98 minutes, with a median of 53 minutes. Following the interviews, the interviewer took notes about the communication setting to support the analysis. All interviews were audio-recorded and transcribed by a research assistant. Background characteristics were collected by self-report, and use data were collected from the national health portal.

To determine whether the number of participants was sufficient, the concept of “information power” was considered [40]. Given the relatively narrow aim of the present study, the limited availability of participants with the appropriate experience (ie, those who had taken part in the randomized controlled trial), and the fact that the interviews generally provided rich data, it was assessed that 15 participants were sufficient.

### Data Analysis

The qualitative content analysis of interviews with caregivers was inspired by Graneheim and Lundman [35]. The process included the immersion in the data, the division into content areas and meaning units, and the condensation of meaning units. The open coding and the abstraction and interpretation of data resulted in categories and subcategories.

To immerse in the data, all transcripts were read several times by the first author (HA). While reading, initial notes were made regarding what was apprehended in the texts. HA also listened to parts of the interviews for recall of the communication environment. During this step of immersion, it was recognized that further analysis would benefit from dividing the text into content areas. This involved extracting statements relating to the different topics covered in the interviews and consolidating them into 3 distinct content areas for the process of coding. During this process, text that was relevant in relation to the purpose of this study was marked and further condensed into meaning units. These meaning units were then coded. The content areas facilitated keeping the context in mind when coding [35]. Codes were further grouped, which then formed subcategories and categories. This step included an abstraction and interpretation while remaining close to the manifest content. The coding and categorization were an iterative process, including going back and forth between content areas, meaning units, and codes, and the full interviews when necessary for making sure that context was kept (Multimedia Appendix 2). This process also ensured “data saturation,” meaning that all data relevant to this study aim were included in the analysis.

In the next step of the analysis, DIEGO was used to identify barriers and facilitators, as well as to formulate strategies to support the scaling up of use.

During the analysis process, coauthors (IT, FA, and AS) read interviews and collaborated in discussions on codes and categories to ensure alignment with participants’ statements and that interpretations stayed close to the interviews. Further, the coauthors collaborated on aligning the caregivers’ statements with the processes and subcomponents of DIEGO [36].

### Ethical Considerations

This study conforms to the Declaration of Helsinki and has been approved by the Swedish Ethical Review Authority (Dnr 2019-05310). Verbal consent was obtained, as participants had previously provided written consent in relation to the main study. During the transcription of interviews, no details that could reveal the identity of the caregiver were included. Participants were offered reimbursement for travel costs but did not receive any other form of compensation. Each interview was initiated with a reminder that participation was voluntary and concluded with an invitation to contact the research team if any questions or concerns were raised as a result of the interview. The data collected is protected in accordance with confidentiality requirements and the safeguarding of personal information.

The Standards for Reporting Qualitative Research [41] were used for preparing this paper (Multimedia Appendix 3).

## Results

### Overview

A total of 15 informal caregivers aged between 52-80 years, most of whom were pensioners, were interviewed. Most caregivers reported having postsecondary education, and most were women who lived with a partner, that is, the family member with HF. About half of the participants stated that they provided support every day. It was most common for caregivers to provide support either daily or weekly, and the most frequently reported types of support were emotional, psychological, or social (Table 1). It emerged from the interviews that the caregivers also received support themselves, for example, from family, neighbors, or health care personnel. However, receiving support from health care was not always self-evident. Further, caregivers could feel lonely in their role, not necessarily due to a lack of support. They also disclosed difficulties in navigating the health care system and making (sometimes tough) decisions on behalf of those they cared for. Earlier caring experiences included feeling forced to provide support and taking responsibility for organizing care.

Usage data collected from the 1177 platform showed that 11 participants had logged on to the support program. Further, 8 of these had accessed at least 4 modules (Table 2).

**Table 1:** Background characteristics and caregiving situation (N=15).

Characteristics		Values
Age (years), range		52-80
**Gender^a^, n**		
	Men	2
	Women	13
**Country of birth, n**		
	Sweden	13
	Other European country	2
**Working status, n**		
	Professional work	6
	Pensioner	9
**Education, n**		
	Compulsory, primary, or elementary	1
	Vocational education	3
	Postsecondary education	11
**Health status, n**		
	Good or very good	9
	Neither good nor bad	6
**Living status, n**		
	Cohabiting	15
	Cohabiting with the person with HF^b^	11
**Relation to the person with HF, n**		
	Partner	11
	Sibling	1
	Parent	1
	Child	2
**How often does the caregiver provide support?, n**		
	Every day	7
	Every week	2
	Less often than once a month	6
**Type of support, help, or care provided, n**		
	Care and treatment	5
	Personal care	1
	Indoor or outdoor mobility	1
	Emotional, psychological, or social	14
	Home and household	10
	Manage finances	8
	Financial support	4
	Organize and manage care, support, or help	5
**Participants’ experience of informal caregiving, n**		
	Not demanding at all	5
	Not very demanding	8
	Quite demanding	2

^a^The question included the option to choose nonbinary or do not wish to answer. We only report men or women because all participants marked either or.

^b^HF: heart failure.

**Table 2:** Participants’ beliefs about seeking support online and their reported use of the support program.

		Values, n
**Belief that the internet could help support one’s own health**		
	Yes	5
	Maybe	6
	No	4
**Belief that the internet could facilitate providing care or support**		
	Yes	3
	Maybe	9
	No	3
**Belief that the internet could help in keeping in contact with health care personnel**		
	Yes	8
	Maybe	6
	No	1
**Logged on to the support program^a^**		
	Yes	11
**How many modules have caregivers accessed^a^**		
	0	5
	1-3	2
	4-15	8
**Time spent on the support program**		
	No time	6
	2 hours or less	3
	More than 2 hours	6
**How many times has the caregiver logged on to the program?**		
	Have not logged in at all	6
	Have logged in 1-4 times	4
	Have logged in more than 4 times	5

^a^Data is collected from the national health portal 1177.

### Categories and Subcategories

As outlined in Textbox 1, the findings from the interviews comprised 2 categories and 6 subcategories and reflected informal caregivers’ experiences of an online support program.

**An overview of categories and subcategories.**

**Engagement was influenced by the macro-, meso-, and microcosm:**

- To be online is timely
- The delivery of the intervention matters
- Personal motives provide reasons
- Happenings in life always impact

**Usage could lead to individual benefits and contribute to the common good:**

- Content provided insights, preparedness, and validation
- Caregivers may not be the only beneficiaries

### Engagement Was Influenced by the Macro-, Meso-, and Microcosm

Caregivers’ engagement with the support program related to the online format, situated on a national health portal (macrocosm), was affected by factors related to the delivery of the intervention (mesocosm) and shaped by personal motives and circumstances that influenced their engagement with the program (microcosm). Respectively, the macro-, meso-, and microcosm reflect societal, organizational, and individual dimensions.

#### To Be Online Is Timely

To be online is timely overrode whether caregivers had engaged with the program, and caregivers expressed that it was appropriate for the support program to be situated online. It was an advantage that the program was accessible through the national health portal, which increased accessibility and credibility. Caregivers found it unproblematic to log into the support program, even though some had initially thought it would be difficult. They were aware that technical support was available, but it was scarcely used.

The electronic identification had not been a hindering factor for using the support program because they used it for other things in their daily lives. Using an electronic identification could also bring a sense of safety.

The participants received an email with instructions on how to log in to the support program and on how to navigate it. Their experience of the instructions ranged from finding them useful to unhelpful. It was suggested that it might be sufficient to provide written instructions solely for logging in, while leaving out the instructions on navigating the support program. Many caregivers could rely on experiences with the national health portal.

#### The Delivery of the Intervention Matters

The delivery of the intervention matters included perceptions about user reminders, the time limit for accessing the support program, and participants’ experiences of the usefulness of the support program.

The reminders were considered to give “a little push,” and reminders could influence caregivers to get started and push the support program onto the caregivers’ agenda. Reminders were even described as crucial for usage: “Honestly, I did it because I received reminders via email, you know.” Experiences with reminders as triggers for usage could depend on how the research group provided them. A personal contact could be motivating and promote the feeling of being included. Telephone calls were also perceived as valuable when the purpose of the contact extended beyond conveying information. Despite disclosing that all forms of communication were acceptable, caregivers had diverse experiences of their effectiveness depending on the intended purpose. Telephone calls could be perceived as less useful due to the risk of calls coming at inconvenient times, difficulties hearing due to hearing loss, and a general aversion to having them. Furthermore, caregivers expressed that text messaging and emails could be sufficient for conveying information or for making them aware, with the advantage being that caregivers could read messages at their convenience. However, the experience was also that they could be more easily overlooked or ignored and less personal. One caregiver who did not possess a smartphone mentioned that lengthy text messages could be difficult to read.

Caregivers who had not engaged in the support program reasoned that reminders induced a sense of guilt, but this was still insufficient to motivate them to log in:

…it [i.e. reminders] actually encouraged me to want to go in and do it, even though I didn’t actually do it. So, I didn’t take offense at all; rather, I thought it was good.

Not perceiving reminders as triggering for using the support program encompassed not needing it, because they had already logged in before receiving them. Additionally, being reminded of something that one is already aware of could be experienced as impersonal.

Reminders could also provide caregivers with information that they might not have fully understood or remembered from previous communication with the research group. This information involved aspects such as having overlooked the existence of a timeline for accessing the support program or even being unaware of the support program’s existence.

Moreover, having a time limit for access to the support program could have affected the use and usability of the support program. Experiences concerning the time limit varied, and it was reasoned to be affected by the specific period when caregivers gained access, such as summer vacations, which may interfere with and influence engagement. It was noted that a shorter time limit could stimulate use and offer a motivation to go through the whole program, “...if I hadn’t known it had to be completed within 3 months, I probably wouldn’t have looked at everything...” The time limit could also influence the usability of the support program because caregivers stated that unrestricted access could have facilitated seeking information when needed. This also allowed them to go back for information that had been forgotten “...because not everything stays, one would want to have more access to it so that you could go back and look again...”

#### Personal Motives Provide Reasons

Caregivers had both individualistic and family-centered motives for wanting to use the online program. Caregivers described anticipating learning something new and having an interest in acquiring information and knowledge, as well as wanting to update previously acquired knowledge, as motives for accessing the support program. Further, caregivers described wanting to become more prepared. This was linked to either HF becoming more prominent in their lives or to realizing they were unprepared for the emotional impact of living with HF. Feeling prepared was conveyed as being of great importance, as expressed by this caregiver when talking about hoping to become stronger and being able to handle her situation better, “I am alone, I cannot expect my daughter or son to come and help [since not having children] and take over, relieving me, so I need myself 100%.”

Caregivers further explained that a reason for accepting participation was on behalf of the person with HF. It was pointed out that learning about HF would hopefully contribute to them being able to better support their family member.

#### Happenings in Life Always Impact

Happenings in life always impact, including how situations in life affected how caregivers engaged in the support program.

It could be challenging to find the time for engagement related to things happening at work, in a caring situation, or in life in general. In relation to professional work, it was pointed out that things that happened there could affect the motivation to engage online:

...we have had a lot of IT problems [at work], so that could also be a contributing reason, that there has been so much trouble, and you just don’t feel like using yet another programme.

Caregivers described that their situation could make it feel relevant to use the support program, as illustrated by this caregiver:

…it's fantastic for me right now because things are starting to happen within us, our family, and just because he's starting to feel a bit worse [the person with HF], so this came to me perfectly, but before that, no, I haven't thought much about it

referring to support for oneself

Experiences also included less engagement with the support program when the family member’s HF symptoms were mild, with the understanding that engagement could increase if the condition worsened. Other factors that were mentioned relating to use included caregivers’ own health issues, the loss of a family member, and encountering problems with internet access.

### Usage Could Lead to Individual Benefits and Contribute to the Common Good

The support program was described as pedagogical and engaging based on its structure, and it was pointed out that you could access it from wherever it suited you. It could be experienced as time-consuming and extensive, and not everything was read by everyone, but caregivers highlighted the broad range of topics, noting its usefulness for both new and long-term caregivers, regardless of their role or tasks. No content was avoided, and the program did not evoke unwanted feelings, although some parts were described as emotional. The content was also described as having “a lot of humanity.”

This category also reflected how usage affected the caregivers themselves and how the support program could have a broader use and be of value for others as well.

#### Content Provided Insights, Preparedness, and Validation

Content provided insights, preparedness, and validation encompassed the perception that the support program offered caregivers diverse and positive experiences.

Caregivers described that using the support program provided them with knowledge and insights, or an update on pre-existing knowledge. When talking about whether the support program had met their prior expectations, 1 caregiver said, “Yes, but that’s what I think and more than that. I have to say, yes, because I also learned things that I don’t think I’ve considered…” Caregivers noted concrete examples, for example, of having gained insight into the varying experiences of persons with HF and their caregivers, as well as having developed a deeper understanding of the severity of HF, “...what I didn’t have knowledge about was actually how serious heart failure is...”

Engaging with content also brought experiences of having received confirmation, “…there are others who have the same experience as me…” and caregivers described this as empowering. The content also provided caregivers with reassurance in their decision to continue making space for themselves, even if the person with HF’s condition deteriorates. Caregivers felt that the content contributed to them feeling acknowledged and that it confirmed their thoughts and actions as caregivers. This recognition contributed to feelings of being important.

Caregivers described becoming prepared after using the support program. Being prepared included having information about what the future may hold for someone with HF and having made practical preparations in relation to the caregiving situation. These experiences also integrated setting aside the knowledge until it becomes relevant or necessary to apply. Caregivers also conveyed that the support program had offered a sense of security and expressed feeling more at ease knowing more about HF and having made preparations.

The content in the support program could also spark new questions or thoughts in relation to HF and in relation to being a caregiver, “…maybe I should be more worried, but I feel that it doesn’t help me... and it might be very cold of me, I don’t know.” These thoughts or questions also included thinking about whether the earlier lack of knowledge might have made for unnecessary comments about encouraging the family member to do things they might not be able to do due to HF.

Engaging with the content also led caregivers to take more actions for their own well-being and helped them better understand their situation. Additionally, some caregivers felt “lucky” when they realized that the content reflected situations worse than their own.

Even though the experience of engaging with the program seemed to have been positive, caregivers also highlighted that they may not necessarily need all the content. In connection with such statements, caregivers pointed out that this could be because their care recipients were not as ill as those featured in the support program. However, caregivers may still consider the content valuable for the future. Some caregivers also mentioned that, while they found the content potentially valuable, it might not entirely replace the importance of having direct interactions with health care professionals.

#### Caregivers May Not Be the Only Beneficiaries

Caregivers described that the support program could benefit other people as well as themselves, suggesting its broader relevance beyond informal caregivers. They described how the support program was helpful in supporting their family member and noted that they had gained a better understanding of them, which was perceived as beneficial for the person with HF. It was also suggested that caregivers’ use of the support program had an impact on the person with HF, “I feel calmer, and of course, he does too naturally then.” Furthermore, as a result of engaging with the support program, caregivers became more observant of changes in the family member. Knowing the symptoms could enhance the ability to ask relevant questions to the family member and help the caregiver in assisting them in seeking medical care if needed.

When describing the broader use of the support program caregivers especially pointed out the value of other family members engaging with the content to enhance their understanding of the situation, “…this programme would be useful for the children to read as well, you know…” further emphasizing that it could relieve the caregiver from the responsibility of reporting on the partner’s condition, as well as enhancing the children’s understanding of the caregiver’s situation, “…I think they have some understanding, but not the full extent of understanding, you know, about the problem….”

Caregivers described that it could also be of value for the person with HF to engage with the content in the program. Caregivers were not very specific in these descriptions, but mentioned that there were parts they wished that the person with HF could read, and that this might lessen the need for caregivers to repeatedly remind them about things.

Moreover, caregivers also conveyed that the knowledge and answers retrieved from the support program could relieve some of the health care burden. It was noted that the support program could serve as a first-line support for informal caregivers, promptly addressing their specific inquiries.

### Results From Using DIEGO

To reflect on potential actions for scaling up use, we identified barriers and facilitators based on caregivers’ experiences and mapped these onto a modified version of DIEGO [36], in order to illuminate factors that may influence caregivers’ decisions and actions regarding engagement with the online support program. These barriers and facilitators were identified in relation to subcomponents of DIEGO, which correspond to the model’s 4 processes. These processes, in turn, relate either to the decision to engage with this type of intervention or to the actual engagement (Table 3).

**Table 3:** Identified barriers (B) and facilitators (F) for engaging with an online support program on a national health portal, incorporating caregivers’ experiences, using the DIEGO^a^.

Process and subcomponents in DIEGO				Identified facilitators (F) and barriers (B)^b^
**These processes are assumed to affect the decision on whether to engage with digital interventions or not**				
	**Making sense of a digital health intervention**			
		Motivation	F: Caregivers can have multiple motives for engaging in a support program online. B: Not perceiving a need for support in relation to being an informal caregiver.	
		Awareness and understanding	F: It is timely to go online for support. B: A support program might not be perceived as beneficial by caregivers in their current situation.	
		Personal agency (choice and control).	F: The availability of the support program is convenient. B: (1) A time limit for access may hinder personal agency. (2) Caregivers can have other sources of support, reducing their interest in online sources.	
	**Considering the quality of a digital health intervention**			
		Usability	F: It is nonproblematic to access and navigate the support program. B: Caregivers may not have electronic identification, which could lessen the usability of digital interventions.	
		Quality of digital health information and interactions	F: (1) The content in the support program can provide positive experiences, and caregivers can feel both prepared and validated when engaging with it, even if they do not expect it. (2) The co-designed content can add similar value to interactions with health professionals and peers and could also be valuable for others besides the primary caregiver. (3) Previous negative experiences with “offline care” could push the use of online solutions.	
**These processes are assumed to affect whether or not a person actually engages with the digital intervention**				
	**Gaining support for enrolling in a digital health intervention**			
		Clinical endorsement	No data	
		Personal advice	No data	
		Direct support	F: Support to handle technology (ie, the platform for the online support) is not necessarily needed.	
		Recruitment strategy	B: Information concerning the digital intervention provided by the research group may not always be understood as intended, and caregivers have different opinions on what constitutes sufficient information.	
	**Registering for a digital health intervention**			
		Security and privacy	F: Logging into 1177 with electronic identification to use the support program is experienced as a safe environment.	
		Skills and equipment	F: Instructions for logging in and using the support program can be helpful. B: (1) Bad internet access. (2) The current version requires logging in using electronic identification, which not everyone may have.	
		Personal lifestyle	F: Caregivers are already using 1177 in their interactions with health care. B: (1) The support program can be experienced as time-consuming and extensive, and caregivers may lack the time and energy to engage online due to, for example, their work or caring situation. (2) Caregivers could prefer physical meetings.	

^a^DIEGO: Digital Health Engagement Model.

^b^Adjustments to the facilitators and barriers in the Digital Health Engagement Model have been made to fit this study’s digital health intervention, that is, an online support program with no personal interaction.

Subsequently, the identified barriers and facilitators were used to reflect on aspects that could support the scaling up of the support program (Multimedia Appendix 4). Two subcomponents, namely “clinical endorsement” and “personal advice,” were not reflected in caregivers’ experiences. However, based on the recommendations provided in DIEGO, these subcomponents were also considered when reflecting on promoting enrollment and use, and furthermore served as the foundation for formulating implementation strategies. The identified strategies were (1) “active outreach,” relying solely on passive dissemination, such as publishing the program online without additional efforts to raise awareness among relevant stakeholders, is unlikely to be sufficient; (2) “supporting endorsement,” which involves identifying trusted sources who can encourage caregivers to engage with the online support program; and (3) “continuous adaptation of the intervention,” incorporating ongoing feedback from users and integrating new evidence, is believed to be important for sustaining long-term use.

## Discussion

Although online interventions hold promise for supporting caregivers, and implementation considerations were integrated into the co-design process that led to the development of the online support program for informal caregivers, challenges related to use persist. It has been emphasized that maintaining caregivers’ engagement can be challenging [16], and one conclusion in relation to our results is that it may entail an ongoing effort over time. This highlights the need to continuously examine how context and intervention interact within specific settings.

This study focused on exploring the experiences of informal caregivers of persons with HF who had access to an online support program and to identify facilitators and barriers in order to reflect on implementation strategies for scaling up use. These insights provided context-specific implementation strategies. Tailoring implementation strategies is recognized as an important aspect of identifying factors that could affect the scaling up of enrollment and use [32]. Using the DIEGO model [36] proved to be useful for this purpose.

### Implementation Strategy 1: Active Outreach

The findings indicate that caregivers have varying motives for using an online support program. Caregivers also described a variation of caregiving situations, including not feeling that HF plays a central role in their lives, which likely affected their engagement with the support program. The absence of personal motives and the lack of perceived burden could influence how these caregivers perceive the importance of using the support program. Even so, our findings indicate that caregivers with different motives and experiences of their caregiving situation described that the content in the support program had positively impacted them in various ways. This could therefore be seen as validating that the support program is suitable for a broad range of informal caregivers of persons with HF. Further, some caregivers took part in the whole program even though not initially feeling the need for it, but rather to comply with what they perceived as expected. These findings align with earlier research [42,43] and could have compensated for the absence of personal motives for some, while also leading to the discovery of the content’s value for them personally.

Engaging with the support program provided caregivers with positive experiences that exceeded being useful only for themselves. Caregivers in our study pointed out several benefits of the support program, and it appeared that acquiring knowledge and insights provided a foundation for other benefits, such as feeling acknowledged by the content. These aspects are important for partner caregivers [44] and may facilitate engagement if caregivers are made aware of how the content can help them recognize themselves in it [22]. If this occurs, it may partially compensate for the lack of interaction with other caregivers, as social support from peers is a crucial source of support [24] and a facilitator for engaging with digital interventions [36].

These different aspects could be of importance to consider when scaling up use, as it may be important to actively approach caregivers [45] using these experiences to motivate them in engaging with an online informal caregiver support.

### Implementation Strategy 2: Supporting Endorsement

O’Connor et al [36] highlighted that endorsement from relevant sources can influence caregivers’ engagement with digital interventions. This could be achieved by targeting health care professionals, thereby increasing the likelihood of clinical endorsements by informing them of the program’s potential. Persons with HF or other family members may also be important for reaching caregivers in need of support*.* Implementation strategies could leverage caregivers’ experiences with the support program as a proxy for “personal advice” from peers, which is recognized as an important facilitator in DIEGO. To reach a broad group of caregivers, it is important to explore relevant information channels, such as persons with HF, health care professionals, and online, print, and social media channels.

### Continuous Adaptation of the Intervention

Adaptation of interventions has been pointed out as an important implementation strategy [32]. Adaptations to the support program could include removing the need to log in using electronic identification. Another factor influencing use highlighted as a potential limitation by the participants was the time limit for accessing the support program. Previous research has underscored the significance of the timing at which interventions are received, pertaining to, for example, participants’ caregiving and life circumstances [42]. A time-limited access could possibly hinder the provision of timely support and information, which needs to be taken into consideration when broadly implementing a support program such as ours.

Additionally, some caregivers pointed out that the support program was extensive and time-consuming, which may be a barrier to engagement in online interventions. This also adds an important aspect for scaling up, as it may be wise to include the opportunity to be guided in choosing topics and content relevant to the caregiver. This can further provide a more personalized intervention, which could facilitate sustained use [22]. Using conversational artificial intelligence, such as a specialized chatbot, could be a feasible option [46,47].

### Additional Aspects to Consider When Scaling Up

The results in this study also point out that the use of reminders and careful consideration of communication with caregivers may be an important factor for maintaining engagement over time. While these strategies were generally appreciated by participants and promoted engagement, as seen in earlier research [22], it is noted that the information and reminders did not prompt all caregivers. Caregivers in our study further highlighted the importance of personal contacts for stimulating use. The support program was developed with considerations for implementation and sustainability [21], which included the intention that the program could be used as independently as possible. However, it may be worth considering continuing with reminders in some form when an intervention such as ours is implemented into routine care.

### Limitations

There are some potential limitations to consider when interpreting the findings of this study, as they could also impact its trustworthiness. Even though there was heterogeneity in relation to, for example, the caregivers’ relationship to the person with HF, working status, and age, most participants were women, born in Sweden, and had a higher level of education. This reflects that most informal caregivers are women who are spouses of the person with HF, and that most people engaging in research are, for example, well-educated [48]. Even so, this raises concerns that should be taken into account. However, we argue that, as the study as a whole included caregivers with shorter educational backgrounds, individuals born outside Sweden, and men, the rich data collected still provide a broad base of experiences. The interviews were performed between 20-115 days after having had access to the support program. This could have made it difficult for some caregivers to recall details about their experiences. Nonetheless, caregivers were able to point out concrete examples and what had lingered, such as the feeling of having been acknowledged in their caring situation through the support.

### Conclusions

The co-design process to develop online support on a platform well-integrated into the Swedish health care system facilitates acceptance and use. However, it has also highlighted potential limitations in aligning fully with caregivers’ preferences on design and features, which may impact broader implementation. By incorporating caregivers’ experiences and using the DIEGO model to identify barriers and facilitators of scaling up use, we illuminated further actions that may promote implementation. Our study validates the DIEGO model [36] as a useful and adaptable heuristic tool that contributes to the broader understanding of scaling up digital health interventions, adding significant value to this field of research. In a health care context, DIEGO could therefore be considered a useful tool for identifying factors that may provide meaningful insight into the implementation process in both clinical practice and research.

## Appendix

Multimedia Appendix 1 The topics and main questions in the interview guide.

Multimedia Appendix 2 Examples of the analytical process, from meaning unit to category.

Multimedia Appendix 3 SRQR checklist.

Multimedia Appendix 4 Reflections on factors that could affect scaling up use.

## Abbreviations

BOTT: burden of treatment theory
DIEGO: Digital Health Engagement Model
HF: heart failure
NPT: normalization process theory

## Data Availability

The dataset generated and analyzed during this study is not publicly available to ensure participants’ confidentiality. However, detailed descriptions of the methods and data analysis, along with some additional descriptive data, can be obtained from the corresponding author upon reasonable request.

## References

[1] (2024). About carers. Eurocarers.

[2] Zigante V (2018). Informal care in Europe: exploring formalisation, availability and quality. European Commission.

[3] European Commission (2021). Long-term care report: trends, challenges and opportunities in an ageing society. European Union.

[4] Charalambous A (2023). Informal Caregivers: From Hidden Heroes to Integral Part of Care.

[5] Savarese G, Becher PM, Lund LH, Seferovic P, Rosano GMC, Coats AJS (2023). Global burden of heart failure: a comprehensive and updated review of epidemiology. Cardiovasc Res.

[6] McDonagh TA, Metra M, Adamo M, Gardner RS, Baumbach A, Böhm M, Burri H, Butler J, Čelutkienė J, Chioncel O, Cleland JGF, Coats AJS, Crespo-Leiro MG, Farmakis D, Gilard M, Heymans S, Hoes AW, Jaarsma T, Jankowska EA, Lainscak M, Lam CSP, Lyon AR, McMurray JJV, Mebazaa A, Mindham R, Muneretto C, Francesco Piepoli M, Price S, Rosano GMC, Ruschitzka F, Kathrine Skibelund A, ESC Scientific Document Group (2021). 2021 ESC guidelines for the diagnosis and treatment of acute and chronic heart failure. Eur Heart J.

[7] Kitko L, McIlvennan CK, Bidwell JT, Dionne-Odom JN, Dunlay SM, Lewis LM, Meadows G, Sattler ELP, Schulz R, Strömberg A, American Heart Association Council on Cardiovascular Stroke Nursing, Council on Quality of Care Outcomes Research, Council on Clinical Cardiology, Council on Lifestyle Cardiometabolic Health (2020). Family caregiving for individuals with heart failure: a scientific statement from the American Heart Association. Circulation.

[8] Choi S, Kitko L, Hupcey J, Birriel B (2021). Longitudinal family caregiving experiences in heart failure: secondary qualitative analysis of interviews. Heart Lung.

[9] Andréasson F (2021). Doing Informal Care: Identity, Couplehood, Social Health and Information and Communication Technologies in Older People's Everyday Lives.

[10] Grant JS, Graven LJ (2018). Problems experienced by informal caregivers of individuals with heart failure: an integrative review. Int J Nurs Stud.

[11] Bangerter LR, Griffin JM, Dunlay SM (2019). Positive experiences and self-gain among family caregivers of persons with heart failure. Gerontologist.

[12] Liljeroos M, Agren S, Jaarsma T, Strömberg A (2014). Perceived caring needs in patient-partner dyads affected by heart failure: a qualitative study. J Clin Nurs.

[13] Gusdal AK, Josefsson K, Adolfsson ET, Martin L (2016). Informal caregivers' experiences and needs when caring for a relative with heart failure: an interview study. J Cardiovasc Nurs.

[14] Hassan AYI, Lamura G, Hagedoorn M (2022). Predictors of digital support services use by informal caregivers: a cross-sectional comparative survey. BMJ Open.

[15] Hassan AYI, Bronzini M, Lamura G (2023). Digital technologies as sources of information for patients and caregivers during COVID-19 pandemic: a cross-sectional survey. Digit Health.

[16] Zhai S, Chu F, Tan M, Chi N, Ward T, Yuwen W (2023). Digital health interventions to support family caregivers: an updated systematic review. Digit Health.

[17] Ploeg J, Ali MU, Markle-Reid M, Valaitis R, Bartholomew A, Fitzpatrick-Lewis D, McAiney C, Sherifali D (2018). Caregiver-focused, web-based interventions: systematic review and meta-analysis (part 2). J Med Internet Res.

[18] Sherifali D, Ali MU, Ploeg J, Markle-Reid M, Valaitis R, Bartholomew A, Fitzpatrick-Lewis D, McAiney C (2018). Impact of internet-based interventions on caregiver mental health: systematic review and meta-analysis. J Med Internet Res.

[19] Hassan AYI (2020). Challenges and recommendations for the deployment of information and communication technology solutions for informal caregivers: scoping review. JMIR Aging.

[20] Andréasson F, Andreasson J, Hanson E (2018). Developing a carer identity and negotiating everyday life through social networking sites: an explorative study on identity constructions in an online Swedish carer community. Ageing Soc.

[21] Allemann H, Andréasson F, Hanson E, Magnusson L, Jaarsma T, Thylén I, Strömberg A (2023). The co-design of an online support programme with and for informal carers of people with heart failure: a methodological paper. J Clin Nurs.

[22] Svendsen MJ, Wood KW, Kyle J, Cooper K, Rasmussen CDN, Sandal LF, Stochkendahl MJ, Mair FS, Nicholl BI (2020). Barriers and facilitators to patient uptake and utilisation of digital interventions for the self-management of low back pain: a systematic review of qualitative studies. BMJ Open.

[23] Berardi C, Antonini M, Jordan Z, Wechtler H, Paolucci F, Hinwood M (2024). Barriers and facilitators to the implementation of digital technologies in mental health systems: a qualitative systematic review to inform a policy framework. BMC Health Serv Res.

[24] Thoits PA (2020). “We Know What They’re Going Through”: social support from similar versus significant others. Sociol Q.

[25] Barbabella F, Poli A, Hanson E, Andréasson F, Salzmann B, Döhner H, Papa R, Efthymiou A, Valenza S, Pelliccioni G, Lamura G (2018). Usage and usability of a web-based program for family caregivers of older people in three European countries: a mixed-methods evaluation. Comput Inform Nurs.

[26] 1177.se This is 1177.

[27] Greenhalgh T, Wherton J, Papoutsi C, Lynch J, Hughes G, A'Court C, Hinder S, Fahy N, Procter R, Shaw S (2017). Beyond adoption: a new framework for theorizing and evaluating nonadoption, abandonment, and challenges to the scale-up, spread, and sustainability of health and care technologies. J Med Internet Res.

[28] WHO (2021). Global Strategy on Digital Health 2020-2025.

[29] WHO (2022). Health and care workforce in Europe. Time to act.

[30] (2022). A European Care Strategy for caregivers and care receivers. European Commission.

[31] (2022). Nationell anhörigstrategi - inom hälso- och sjukvård och omsorg [National Carers Strategy - within Health and Social Care], Promemoria S2022/02134. Regeringskansliet.

[32] Powell BJ, Waltz TJ, Chinman MJ, Damschroder LJ, Smith JL, Matthieu MM, Proctor EK, Kirchner JE (2015). A refined compilation of implementation strategies: results from the Expert Recommendations for Implementing Change (ERIC) project. Implementation Sci.

[33] Eriksson K, Lindström UA (1997). Abduction—a way to deeper understanding of the world of caring. Scand J Caring Sci.

[34] Graneheim UH, Lindgren BM, Lundman B (2017). Methodological challenges in qualitative content analysis: a discussion paper. Nurse Educ Today.

[35] Graneheim UH, Lundman B (2004). Qualitative content analysis in nursing research: concepts, procedures and measures to achieve trustworthiness. Nurse Educ Today.

[36] O'Connor S, Hanlon P, O'Donnell CA, Garcia S, Glanville J, Mair FS (2016). Understanding factors affecting patient and public engagement and recruitment to digital health interventions: a systematic review of qualitative studies. BMC Med Inform Decis Mak.

[37] May C, Finch T (2009). Implementing, embedding, and integrating practices: an outline of normalization process theory. Sociology.

[38] May CR, Mair F, Finch T, MacFarlane A, Dowrick C, Treweek S, Rapley T, Ballini L, Ong BN, Rogers A, Murray E, Elwyn G, Légaré F, Gunn J, Montori VM (2009). Development of a theory of implementation and integration: normalization process theory. Implement Sci.

[39] May CR, Eton DT, Boehmer K, Gallacher K, Hunt K, MacDonald S, Mair FS, May CM, Montori VM, Richardson A, Rogers AE, Shippee N (2014). Rethinking the patient: using burden of treatment theory to understand the changing dynamics of illness. BMC Health Serv Res.

[40] Malterud K, Siersma VD, Guassora AD (2016). Sample size in qualitative interview studies: guided by information power. Qual Health Res.

[41] O'Brien BC, Harris IB, Beckman TJ, Reed DA, Cook DA (2014). Standards for reporting qualitative research: a synthesis of recommendations. Acad Med.

[42] Stjernswärd S, Hansson L (2020). A qualitative study of caregivers' experiences, motivation and challenges using a web-based mindfulness intervention. Community Ment Health J.

[43] de Vries HJ, Kloek CJJ, de Bakker DH, Dekker J, Bossen D, Veenhof C (2017). Determinants of adherence to the online component of a blended intervention for patients with hip and/or knee osteoarthritis: a mixed methods study embedded in the e-exercise trial. Telemed J E Health.

[44] Wang Z, Tocchi C (2023). Partners' experience of informal caregiving for patients with heart failure: a meta-ethnography. J Cardiovasc Nurs.

[45] Hanson E, Charalambous A (2023). Caring for the informal carer: coping in caregiving. Informal Caregivers: From Hidden Heroes to Integral Part of Care.

[46] Lee P, Bubeck S, Petro J (2023). Benefits, limits, and risks of GPT-4 as an AI chatbot for medicine. N Engl J Med.

[47] Skjuve M, Brandtzaeg PB, Følstad A (2024). Why do people use ChatGPT? Exploring user motivations for generative conversational AI. First Monday.

[48] Resurrección DM, Moreno-Peral P, Gómez-Herranz M, Rubio-Valera M, Pastor L, Caldas de Almeida JM, Motrico E (2019). Factors associated with non-participation in and dropout from cardiac rehabilitation programmes: a systematic review of prospective cohort studies. Eur J Cardiovasc Nurs.

