# Supplementary material for: Informal Caregivers’ Experiences of an Online Support Program: Qualitative Study Using an Abductive Approach Focusing on Scaling Up Use
Source: J Med Internet Res. 2025 Nov 27;27:e77576. doi: 10.2196/77576 (PMC12699252; doi:10.2196/77576)
Supplement: Multimedia Appendix 1 [file jmir_v27i1e77576_app1.docx]

| **Topic** | **Questions** |
| --- | --- |
| ***Informal carers’ experiences of their own life situation*** | I would like to hear about your experiences of being an informal carer to a person with heart failure. Would you like to tell me about that? |
| ***Informal carers’ motivations and expectations regarding participation in the randomised controlled trial*** | Why did you decide to participate in the research study? |
|  | If you look back, what expectations did you have for participating in the research project? |
| ***Informal carers’ experiences, perceptions, and motivations for using the support programme*** | Would you like to tell me about *how* you have used the support programme?* |
|  | We in the research group would like to learn more about your experiences and perceptions of the content in the support programme. What would you say the support programme has contributed to you, or provided for you?* |
|  | What do think has influenced your use of the support programme?** |
|  | What do you think about the support programme being online?** |
|  | We‘d like to know how you perceived our contacts with you.. How did you experience the contacts we had with you?** |
| * Only those having engaged with the support programme were asked.  **** Both users and non-users were asked. | |
