# Supplementary material for: Informal Caregivers’ Experiences of an Online Support Program: Qualitative Study Using an Abductive Approach Focusing on Scaling Up Use
Source: J Med Internet Res. 2025 Nov 27;27:e77576. doi: 10.2196/77576 (PMC12699252; doi:10.2196/77576)
Supplement: Multimedia Appendix 2 [file jmir_v27i1e77576_app2.docx]

| **Meaning unit** | **Condensed meaning unit** | **Open code** | **Sub-category** | **Category** |
| --- | --- | --- | --- | --- |
| *“…it’s good to prepare oneself maybe a little mentally, not more than necessary, you know … yes, but it has contributed to a greater understanding of my dad and his situation and how it might look going forward…”* | It [the support programme] has contributed with the insight that it is good to prepare for deteriorations. | Getting prepared | Content provided insights, preparedness and validation | Usage could lead to individual benefits and contribute to common good |
| *"Honestly, I did it because I received reminders via email, you know. ‘You haven’t logged in or it’s been a long time’ [non relevant text excluded]… So, I did the first part, and then I postponed it. Then I received a reminder that ‘it’s closing soon’… in 3 weeks or 2 weeks, or whatever it was. So, I sat down and did it then…"* | Reminders have been crucial for the use of the support programme. | Reminders triggered usage | The delivery of the intervention matters | Engagement was influenced by macrocosm, mesocosm and microcosm |
