# Supplementary material for: Informal Caregivers’ Experiences of an Online Support Program: Qualitative Study Using an Abductive Approach Focusing on Scaling Up Use
Source: J Med Internet Res. 2025 Nov 27;27:e77576. doi: 10.2196/77576 (PMC12699252; doi:10.2196/77576)
Supplement: Multimedia Appendix 3 [file jmir_v27i1e77576_app3.pdf]

Standards for Reporting Qualitative Research (SRQR)*

No.	Topic	Page no.	Comments
S1	Title	Title page	We have endeavoured to align the title with the recommendations in JMIR and SRQR.
S2	Abstract	1	We have aligned the abstract with the given format and strived to include key elements that provide an overview of the study.
S3	Problem formulation	3-4	The aim has been to anchor this project in previous studies and, through current research, provide arguments for why this study is important.
S4	Purpose or research question	Abstract and page 4	
S5	Qualitative approach and research paradigm	5+6	Qualitative approach is outlined in 'Data collection and settings' and 'Data analysis'. Comment on research paradigm not included in the manuscript: However, one of the underlying assumptions in this paper is that a researcher cannot be entirely free from context or prior knowledge, and that there may be multiple interpretations of reality.
S6	Researcher characteristics and reflexivity		Note (not included in the manuscript): The co-authors were experienced in qualitative analysis and had prior knowledge in the fields of heart failure and informal caregiving. We believe it is crucial for aresearcher to be reflexive. Throughout this study, we have engaged in continuous discussions about our position in relation to the data to challenge our assumptions and interpretations. See also S15.
S7	Context	5	In 'Data collection and settings'.
S8	Sampling strategy	5	In 'Data collection and settings'.
S9	Ethical issues pertaining to human subjects	6-7	In 'ethical considerations'

S10	Data collection methods	5	In 'Data collection and settings'. Note (not included in manuscript): For the purpose of this study, we considered individual interviews to be the most appropriate format. We believed this approach would allow for the collection of in-depth data while also protecting participants, many of whom were elderly. The latter was especially important since some restrictions on physical meetings remained in place due to COVID-19 guidelines.
S11	Data collection instruments and technologies	5	In 'Data collection and settings'.
S12	Units of study	5	In 'Data collection and settings'.
S13	Data processing	5-6	In 'Data collection and settings and in 'Data analysis'.
S14	Data analysis	6	
S15	Techniques to enhance trustworthiness	20	In 'Limitations. See also S5+S6. Note (not included in the manuscript): We have been thorough in understanding, describing, and documenting our approaches and methods for data collection and interpretation. We also provide excerpts from interviews to support our findings and interpretations.
S16	Synthesis and interpretation	7-17 + 18-20	In the results section, the synthesis of interviews, and the 'abductive leap' are presented. In the discussion section, the main findings are interpreted in relation to DIEGO and other research.
S17	Links to empirical data	7-9 + 10-16	We provide descriptive data from questionnaires and usage data from the digital platform described in the manuscript. We also include excerpts from interviews in the Results section.
S18	Integration with prior work, implications, transferability, and contribution(s) to the field	18-21	In the Discussion section, 'Limitations' and Conclusion
S19	Limitations	21	

S20	Conflicts of interest		Stated during the submission process.
S21	Funding		In "Title page".

*Created by H Allemann from Table 1 in: O'Brien, Bridget C. PhD; Harris, Ilene B. PhD; Beckman, Thomas J. MD; Reed, Darcy A. MD, MPH; Cook, David A. MD, MHPE. Standards for Reporting Qualitative Research: A Synthesis of Recommendations. Academic Medicine 89(9):p 1245-1251, September 2014. | DOI: 10.1097/ACM.0000000000000388
