# Supplementary material for: Informal Caregivers’ Experiences of an Online Support Program: Qualitative Study Using an Abductive Approach Focusing on Scaling Up Use
Source: J Med Internet Res. 2025 Nov 27;27:e77576. doi: 10.2196/77576 (PMC12699252; doi:10.2196/77576)
Supplement: Multimedia Appendix 4 [file jmir_v27i1e77576_app4.docx]

Reflections on factors that could affect scaling up use, using the identified barriers and facilitators (Table 4 in the manuscript) and *recommendation* 1-5 by O’Connor et al [36] (2016) in the DIgital Health EnGagement MOdel (DIEGO).

| Process(es) | Making sense of a digital health intervention  &  Considering the quality of a digital health intervention | Gaining support for enrolling in a digital health intervention | Registering for a digital health intervention |
| --- | --- | --- | --- |
| Reflections on factors that could affect scaling up use | Since carers may not self-evidently identify as being in a caring role and have different motives for engaging with online content, and as implied by carers in this study, still benefit from engaging with the programme, we should actively reach out to carers to *market* the qualities of the support programme and how an online source such as ours could be beneficial.  To enhance the chances of scaling up usage and reaching those who could benefit from the online content, we should consider removing the need to log in using an electronic identification.  We should also consider removing the specific time frame for usage. Allowing carers to access the programme over a longer period could increase carers’ ‘*sense of personal agency’* as well as the programme’s ability to provide timely support, thereby improving its perceived usability.  (DIEGO - recommendations 1 & 2). | The experiences of informal carers with the support programme can serve as a proxy for ‘*personal advice’* from peers when raising awareness about the programme to potential users. The focus should not only be on carers but also on those who may influence carers’ choice to engage with online support. Even though our findings indicate that carers may not need support to access and use the programme, it is important to highlight that technical support is available for those who might feel insecure about using digital solutions. When scaling up, it is crucial to adhere to different users’ needs and preferences for (recruitment) information. We should employ an approach that ensure that information is useful and not perceived as impersonal. Artificial intelligence (AI) could be valuable in this regard, helping to address these perspectives while still minimising the involvement of healthcare professionals.  To enhance the chances of a successful scaling up, we should include strategies to target healthcare professionals, to help them feel comfortable in ‘*clinically endorsing’* the support programme. Factors that can help health care professionals endorse and promote usage are: 1. The online health portal 1177 is already well-known and used by healthcare workers. 2. Our support programme is mostly self-administered, especially if we exclude the need for electronic identification. This means that endorsing the programme should not add workload. However, we also identify a potential barrier. Even though professionals may consider carer support within their role, organisational factors such as lack of time may, 1) hinder an expanded carer perspective, and 2) make it easy to forget to inform carers due to other pressing work duties.  (DIEGO - recommendations 3 & 4). | Even though usage is logged, it is expected that most will feel comfortable and secure using the support programme on 1177, as it is a well-known and trusted platform regulated by general laws within Swedish healthcare. However, since there is no current reason for healthcare to have access to carers’ usage data, this may provide further support for excluding the need to log in using electronic identification. Ensuring privacy as well as ease of access could enhance the overall user experience and encourage more carers to utilise the support programme.  To accommodate the diverse lifestyles and life situations of carers, which includes perceiving the support programme as time-consuming and extensive, we should seek opportunities to tailor the content to the specific needs of the users. AI could be an option to enhance the ability to personalise the digital intervention.  (DIEGO - recommendation 5). |

Reference:

[36] O'Connor S, Hanlon P, O'Donnell CA, Garcia S, Glanville J, Mair FS. Understanding factors affecting patient and public engagement and recruitment to digital health interventions: a systematic review of qualitative studies. BMC Med Inform Decis Mak 2016; 16(1):120
